# Hidden Coronary Atherosclerosis Assessment but Not Coronary Flow Reserve Helps to Explain the Slow Coronary Flow Phenomenon in Patients with Angiographically Normal Coronary Arteries

**DOI:** 10.3390/diagnostics12092173

**Published:** 2022-09-08

**Authors:** Carlo Caiati, Fortunato Iacovelli, Giandomenico Mancini, Mario Erminio Lepera

**Affiliations:** Institute of Cardiovascular Disease, Department of Emergency and Organ Transplantations, University of Bari “Aldo Moro”, 70124 Bari, Italy

**Keywords:** slow coronary flow, coronary artery disease, intravascular ultrasound, coronary blood flow, coronary flow reserve, coronary angiography

## Abstract

The significance of the slow coronary flow phenomenon (SCFph), as visualized in patients (pts) with angiographically normal coronary arteries, is controversial. Absolute coronary flow reserve (CFR) in the left anterior descending coronary artery (LAD), non-invasively assessed by a transthoracic color-guided pulsed-wave Doppler (E-Doppler TTE), is a reliable parameter to assess coronary microcirculatory dysfunction (CMD). Mild and angiographically hidden epicardial atherosclerosis (Hath), as visualized by intracoronary ultrasound (IVUS), which could be the clue to atherosclerotic coronary microvascular involvement, has never been investigated together with CFR in patients. This study was aimed at assessing the value of CFR and HA in explaining the SCFph. Methods. Both non-invasive assessment of CFR in the LAD and corrected TIMI frame count assessment of the coronary contrast runoff were performed in 124 pts with angiographically normal coronary arteries. Among the whole group, 32 patients also underwent intracoronary ultrasounds in the LMCA and LAD, and the maximal plaque burden was assessed (Lesion external elastic (EEM) cross sectional area (CSA)—Lesion Lumen CSA/Lesion EEM CSA * 100). We found that 24 of the 124 pts (group 1) had the SCFph and the remaining 100 had a normal runoff (group 2). CFR, evaluated in both groups, was not significantly different, being 2.79 ± 0.79 (Mean ± SD) in group 1 and 2.90 ± 0.8 in group 2 (*p* = ns); in the pts also examined by IVUS (32 pts), the SCFph was always associated with hidden atherosclerosis, and a plaque burden of ≥33%. On the contrary, in the normal runoff group, any grade of PB was observed (from no athero to a PB > 70%) and remarkably, 10 pts had no signs of athero or just a minimal plaque burden. This resulted in a ROC curve analysis in which PB < 33% had a high negative predictive value (100%) in ruling out the SCFph. In addition, considering a CFR value < 2.21 as an index of coronary microcirculatory dysfunction, we found CMD in 15 pts (15%) in group 1 and in 7 pts (29%) in group 2 (*p* = ns). In conclusion, the SCFph is strongly connected to epicardial athero to the extent that the absence of hidden coronary athero has a very high negative predictive power in ruling out SCFph. CFR that is based on an endothelium-independent mechanism remains fairly normal in this condition. An endothelium-dependent microcirculatory constriction at rest due to atherosclerotic involvement of the coronary microvascular network is a possible explanation of the SCFph.

## 1. Introduction

The slow coronary flow phenomenon (SCFph) observed during coronary angiography (CA) [1] features a slow contrast runoff in the epicardial coronary arteries (involving one or more coronary artery ) in subjects without lumen narrowing. It is a relatively frequent finding, having an incidence of 1–7% [2].

However, the primary coronary abnormalities influencing this phenomenon are not clear. Microcirculation dysfunction is certainly implicated, but the kind of microcirculatory dysfunction has not been properly elucidated. Even less clear are the causes of this phenomenon involving the microcirculation. The evaluation of coronary flow reserve (CFR), which tests a prevalent microcirculatory endothelium-independent mechanism, yields contrasting results and the data are sparse. Previous authors have demonstrated by invasive assessment that patients with the SCFph have a preserved coronary flow reserve (CFR) despite high microvascular resistances at rest [3]. In another study, CFR results were lower in SCFph patients [4]; however, in that study, an improper vasodilation protocol (standard dose of dipyridamole) was adopted. In fact, in a landmark study, coronary vasodilator potency for dipyridamole, assessed by a coronary vascular resistance index which incorporates changes in arterial pressure, was lower than for adenosine and papaverine [5]. 

Even more importantly, what are the mechanisms causing microcirculatory dysfunction? It is not clear whether hidden atherosclerosis in the epicardial conduit can be in some way the sentinel of the atherosclerotic microcirculatory involvement underlying the SCFph. 

CFR evaluated by enhanced transthoracic Doppler echocardiography (E-Doppler TTE) in the distal left anterior descending coronary artery (LAD), is a very robust parameter previously largely validated with excellent reproducibility and intra–interobserver variability [6,7,8]. 

Therefore, this study was aimed at understanding firstly whether CFR, as assessed by the E-Doppler TTE along with appropriate vasodilation, is lower in SCFph patients compared to subjects with a normal runoff, and secondly, whether hidden athero, as detected by intra-coronary ultrasound (IVUS) in the epicardial conduit, is associated with the SCFph. For this purpose, 124 subjects with angiographically normal coronary arteries (24 showing the SCFph) were enrolled.

## 2. Materials and Methods

The inclusion criteria were as follows: a total of 124 consecutive unselected patients scheduled for a non-invasive Doppler recording of blood flow in the left main coronary artery (LMCA) and the whole LAD along with coronary flow velocity reserve in the distal LAD by E-Doppler TTE, then referred for coronary angiography, whose coronary arteries resulted angiographically normal. Exclusion criteria were: age less than 18 years, asthma, sick-sinus syndrome, II or III atrio-ventricular blocks, long QT syndrome, hypotension, and heart failure. All patients underwent both non-invasive assessment of CFR and TIMI frame count evaluation of coronary contrast runoff in the LAD. 

Non-invasive assessment of CFR. Patients refrained from consuming coffee, tea, and chocolate for at least 48 h before the examination. Each patient underwent a color-guided pulsed-wave Doppler recording of their coronary blood velocity in the distal part of the LAD in the baseline condition and after Adenosine administration (140 mcg/Kg/min over 3 min). Echocardiography was performed using an Acuson Sequoia™ ultrasound unit (C256 Echocardiography System, Siemens Healthcare, Erlangen, Germany) and broadband transducer (3V2c). The Color Doppler signal was attained in convergent color Doppler mode at 2.5 or 2.0 MHz transmission frequency, while Spectral Doppler was performed in fundamental mode at 2.5 or 2.0 MHz [9]. The color-coded Doppler setting was adjusted to maximize scanning sensitivity (pulse repetition frequency was set at 16 cm/s (2.5 MHz) or 20 cm/s (2.0 MHz) with minor modulation in special cases, and the sample volume of color flow mapping was maximized) without significantly reducing the frame rate (the color box size was reduced to keep up with a frame rate of >30 Hz). All results were digitally stored on the built-in dedicated hard drive.

Color Doppler detection of LAD flow was obtained in the distal or in the midpart of the LAD, as already described [6,8]. Briefly, a modified foreshortened two-chamber view was obtained by sliding the transducer substantially and medially from an apical two-chamber position. Then, a careful search for color-coded blood flow was performed over the epicardial part of the anterior wall, trying at the same time to optimize the visualization of the anterior groove area by rotating very slightly counterclockwise and medially angling the probe. 

To measure coronary flow velocity, first, color-coded flow imaging was attained, and then pulsed-wave (PW) Doppler recording was performed, using the color flow as guide. The gate size was set at 4.0 mm. If the angle between the color flow and Doppler beam was higher than 20°, angle correction was performed using the software package included in the ultrasound unit. Sample volume positioning was performed, taking into account the diastolic position of the vessel [6]. The spectral trace of coronary flow velocity was characterized by a biphasic flow with a prevalent diastolic component. 

Coronary flow reserve study protocol. A blood flow Doppler recording in the LAD was first attempted at baseline before infusing intravenous adenosine (140 g/kg per min over 3 min) using an intravenous line. Recording of hyperemic flow velocity by the PW Doppler was started as soon as the color signal showed any velocity increase (brighter color or the appearance of aliasing), or in any case, within 2 min from the beginning of the drug administration, and then continued until the end of the third minute. If during adenosine infusion the theta angle or LAD segment appeared different than that visualized at baseline, a new baseline velocity curve was recorded in the recovery phase. However, this event was almost never observed.

Coronary flow reserve measurement. One experienced echocardiographer who was blinded to the angiographic results performed the blood flow velocity measurement. Measurements were made off-line using the built-in calculation package of the Acuson Sequoia ultrasound unit. 

The coronary flow velocity parameters measured before and during hyperemia in the subgroup of patients undergoing CFR assessment were the peak and mean diastolic velocities. For each parameter, the highest of three (in cases of sinus rhythm: 122 patients) or six cycles (in cases of atrial fibrillation: 2 patients) was averaged. Coronary flow reserve was calculated as the ratio of hyperemic to basal peak (peak CFR) and mean (mean CFR) diastolic flow velocity. 

Coronary angiography and IVUS. All the patients underwent coronary angiography through the trans-femoral or trans-radial route, depending on the physician’s judgement and the patient’s anatomy and clinical condition. 

All angiographic studies were performed and interpreted blinded, since they were performed as routine studies. The coronary stenosis was visually assessed based on multiple projections by one investigator who was unaware of the TTE Doppler results. The presence of minimal luminal irregularities was specifically looked for. 

**Figure 1:**
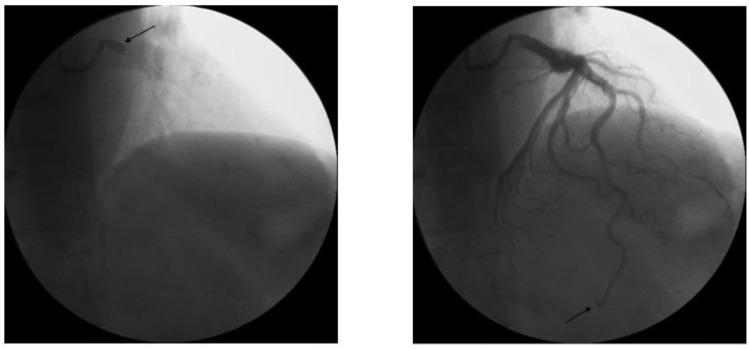
TIMI frame counting method specifically applied to the (**left**) anterior descending artery (LAD). Image on the left (arrow) is the first chosen frame (when LAD ostium is first opacified by contrast) while the image on the (**right)** is the last frame (when the distal LAD is opacified as indicated by the arrow).

For the coronary dye progression, two independent observers that were blinded to the results of CFR calculated the modified TIMI frame count (TFC) in the LAD, assessing the number of frames necessary to opacify the distal LAD in anterograde motion, starting with the frame in which the dye touched both borders of the origin of the vessel and ending where the dye reached the landmark as previously described [10] and reported in the example (Figure 1).

Since the distance between proximal and distal bifurcation in the LAD coronary artery is longer than in other coronary arteries, LAD TFC is significantly higher than the TFCs in the right coronary artery (RCA) and circumflex (LCx) artery. Thus, a correction with the 1.7 constant coefficient was used to standardize the LAD measurements: the non-corrected LAD mean value (±SD) in normal arteries is 36 ± 1; if the frame rate is 30 frames/sec [10], the corrected mean value (±SD) is 21 ± 3 and the normal range value is 2 standard deviations from the mean. Centricity Cardiology (C.A. 1000 2.0, GE Healthcare) software was used for the calculation of TFC.

The vessel diameters were analyzed using a computerized QCA analysis system (QAngioXA, Medis Suite XA, Leiden, The Netherlands). To determine the actual width of the coronary artery lumen, a calibration was conducted using the catheter diameter. The indexed coronary artery diameter has been defined in diastole for each segment as the mean coronary diameter divided by the BSA of the patient. Moreover, assuming a round cross section, the lumen cross-sectional area was estimated to be (π/4)(mean diameter)^2^. The summed cross-sectional area of proximal RCA, proximal LAD, and proximal LCx branches was calculated and, per MacAlpin [11], was called the total coronary area (TCA).

*IVUS* After preventing arterial spasms by administering intracoronary nitroglycerine, an IVUS examination of the LAD was proposed. The exclusion criteria included anatomic situations in which luminal diameter and/or the site and extent of vascular disease precluded the introduction of the IVUS catheter (i.e. very tight stenosis, diffuse atherosclerosis—in which a normal or near-normal segment of artery could not be identified adjacent to a diseased segment); major tortuosity or significant myocardial bridges in order to avoid the dissection or destabilization of the plaque itself; diffuse or isolated coronary spasms; wrinkling or invagination of angled segments by the guidewire, etc.

The total length of the digital IVUS catheter (Eagle Eye Platinum, Volcano Corporation, Rancho Cordova, CA, USA) is 150 cm and it has a transverse profile of 3.5 F at the transducer. The nominal transducer center frequency is 20 MHz (free of non-uniform rotational distortion and guidewire artefacts) and it focuses a maximum imaging diameter of 20 mm. It is compatible with 0.014″ or smaller guidewires, and the probe/wire combination was accommodated in a 6 F guide catheter.

After advancing the IVUS catheter under fluoroscopic guidance to the distal segment of the LAD, the probe was withdrawn at a speed of 1 mm/sec using a disposable pullback device (Trak Back II, Volcano Corporation, Rancho Cordova, CA, USA) until it came out of the LMCA. The procedure was monitored online using one monitor positioned opposite the operator and another mounted on the control panel and was checked by the US technician. The IVUS data were stored digitally and assessed offline using dedicated arterial analysis software (Volcano s5iTM Imaging System, Volcano Corporation, Rancho Cordova, California, USA): all the computer-derived measurements of the digitized images had already been converted into millimeters.

*Analysis of IVUS data* In accordance with the general angiographic definition, the LAD was subdivided into three segments: the proximal segment included the first major septal branch or the first diagonal; the middle segment was immediately distal to the origin of the first diagonal branch and extended to the branch-off point of the last diagonal. 

The presence of plaque in each LAD segment was first qualitatively assessed by detecting atheroma; identifying the tightest stenosis in each segment; and roughly estimating plaque extension (single or multiple) and its possible encroachment into the lumen, ulceration, dissection, or intraluminal thrombosis. The main quantitative analysis consisted of the following measurements: plaque burden, calculated as plaque and media cross-sectional area (CSA) divided by external elastic membrane (EEM) CSA in each of the 4 segments of the left coronary artery examined (left main, proximal, mid, and distal LAD). The plaque and media cross-sectional area was calculated as EEM CSA minus lumen CSA [12]. 

*Statistical analysis* Student’s *t* test was used to compare the continuous variables. The categorical variables were compared by a chi-squared test. The plaque burden value to best discriminate patients with and without SCFph was empirically estimated using receiver operating characteristic (ROC) curves, and bootstrapping (5000 resampling) was used for the 95% CI assessment. We calculated the sensitivity and specificity. A *p* value of <0.05 was considered significant. The data were analyzed using the SPSS 21.0 statistical package for Mac (SPSS Inc., Chicago, IL, USA).

## 3. Results

We found that 24 of the 124 patients (19.4%) (group 1) had the SCFph and the remaining 100 (81%) had a normal runoff (group 2); in the two subgroups, the corrected TIMI frame counts were 35 ± 4 and 19 ± 3, respectively (*p* < 0.0001).

A coronary dilation was not associated with the SCFph group. The coronary dimension, in fact, expressed as proximal LAD diameter, Total Coronary Area, and Indexed Total Coronary Area, was fairly normal and not different in the SCFph group from the control group, being 2.90 ± 0.63 vs. 2.92 ± 0.46 mm (*p* = ns), 18.95 ± 6.28 vs. 18.96 ± 5.27 mm^2^ (*p* = ns), and 9.72 ± 3.17 vs. 9.82 ± 2.55 mm^2^, respectively (*p* = ns). 

**Figure 2:**
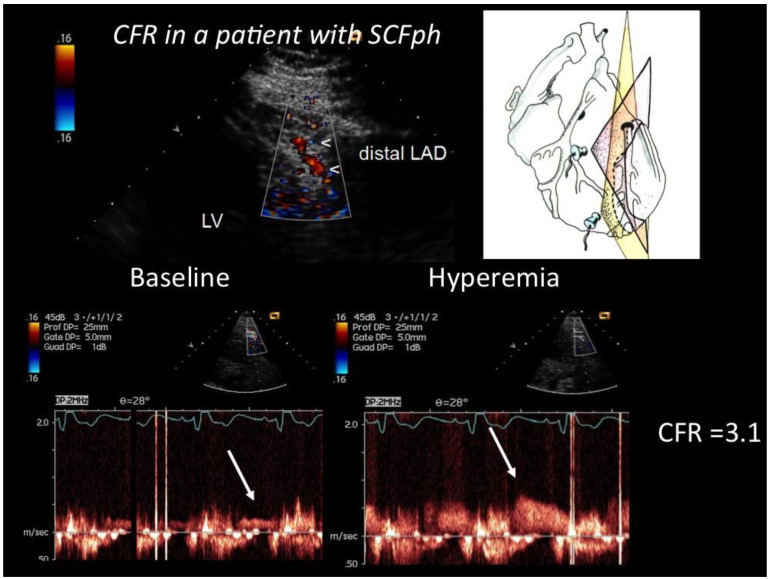
CFR in the distal LAD assessed by E-Doppler TTE in a patient with SCFph. At the **top**, color flow in the distal LAD (in red); on the **right**, a cartoon of the tomographic plane orientation to obtain the LAD insonification. At the **bottom**, pulsed Doppler spectral tracing of the blood flow velocity in the distal LAD at baseline (**left**) and at maximal Adenosine-induced hyperemia (**right**); note the prevalent diastolic BF velocity, a peculiarity of coronary flow. The CFR (peak hyperemic diastolic velocity/peak resting diastolic velocity) is above 3, indicating no significant stenosis and a fairly normal endothelium-independent microcirculatory function.

Coronary flow reserve. An example of how CFR was performed by E-Doppler TTE in a subject with SCFph is shown (Figure 2).

**Figure 3:**
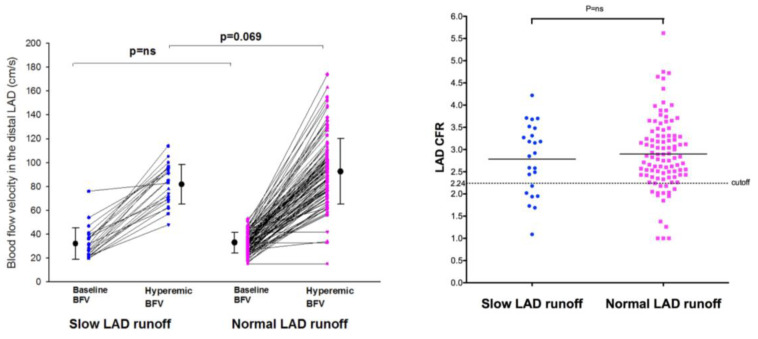
Individual value bar graph of velocities and coronary flow reserve as assessed in the distal LAD in the SCFph group and in the control group. CFR was similar in the SCFph group with respect to the control (graph on the **right**). The hyperemic BFV, however, (graph on the **left**) tended to be a little bit lower in the SCFph patients than in the control, albeit this did not reach a statistical significance; mean values and SD are also drawn. BFV = blood flow velocity; CFR = coronary flow reserve; LAD = left anterior descending coronary artery; SD = standard deviation.

The CFR evaluated in both groups was not significantly different (Table 1 and Figure 3), being 2.79 ± 0.79 (Mean ± SD) in group 1 and 2.90 ± 0.8 in group 2 (*p* = ns), even though the hyperemic velocities tended to be lower in the SCFph group with respect to the controls, but that showed only a statistical trend (*p* = 0.069). 

In addition, considering a CFR value ≤ 2.24 as an index of coronary microcirculatory dysfunction based on a prevalent endothelium-independent mechanism, explored by adenosine as previously validated [13], we found CMD in 15 (15%) group 1 patients and in 7 (29%) group 2 patients (*p* = ns) (Figure 3). Accordingly, the calculated sensitivity and specificity of SCFph in predicting CMD by adenosine were poor: 29% (6/31) and 85.3% (40/48) (*p* = ns), respectively (Figure 3). The SCFph was significantly associated with coronary risk factors such as LDL cholesterol; moreover, smoking was more frequent in the SCFph group than in the controls, but without reaching a statistical significance (Table 2).

**Figure 4:**
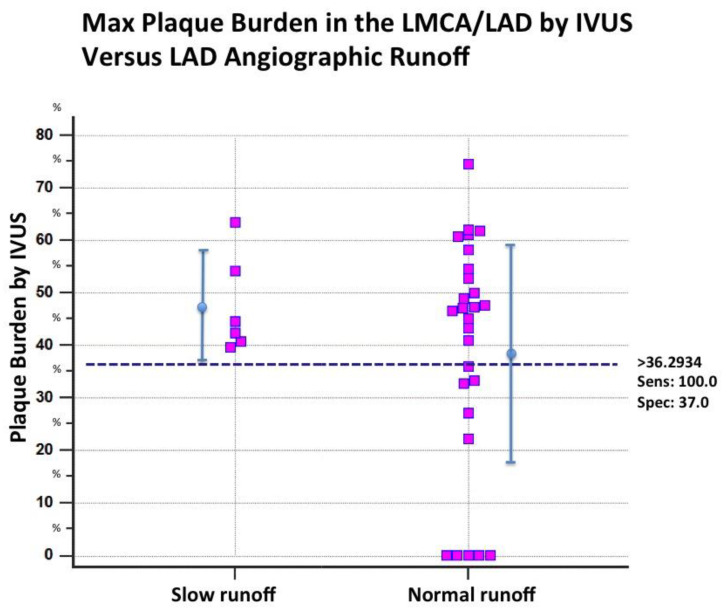
Individual value bar graph of plaque burden in the patients with slow and normal contrast dye progression (subgroup of 32 patients). In the SCFph group, plaque burden was at least moderate, and all patients had a hidden athero; in contrast, in the control group, a large part of the subjects had no athero at all or minimal athero. By ROC analysis, a PB value < 38% had very high negative predictive value (100%) in ruling out hidden athero. Sens = sensitivity; Spec = specificity; IVUS = intravascular ultrasounds; LAD = left anterior descending coronary artery; LMCA = left main coronary artery.

Evaluation of hidden atherosclerosis. In a subgroup of 32 patients (six with the SCFph) in which IVUS in the LMCA and LAD was performed, hidden atherosclerosis was found in most of them (22 patients), while in 10, the atherosclerosis-related plaque burden was either totally absent (no atherosclerosis visualized) (five patients) or sparse and minimal (five patients). To evaluate the extension of hidden athero, we subdivided the LMCA and LAD into four segments: one segment included the LMCA and three segments the LAD (proximal, mid, and distal segments); most of the patients (22 patients, 67%) had extensive hidden atherosclerosis with at least two segments involved. The maximum plaque burden was 47.48 %± 9.04 in the SCFph group and 38.5 ± 21% in the normal runoff group (*p* = ns). An evaluation by the ROC curve analysis showed that in the slow runoff group, all patients had hidden athero and a minimal PB of 36%. A PB cutoff < 36% (bootstrapped CI 95% < 32.77 to <61.78) showed a very high negative predictive power (100%) in ruling out the SCFph (Figure 4). Therefore, absent or minimal athero, as assessed by the PB, was a condition that ruled out the SCFph. The presence of epicardial athero was a prerequisite for the SCFph, indicating that epicardial athero can extend into the microcirculation in some cases, causing athero-related microcirculation dysfunction in the form of abnormal arterioles vasoconstriction. 

## 4. Discussion

The major finding in this study is that with an appropriate vasodilation stimulus (adenosine), the CFR is shown not to be reduced in patients with the SCFph; and that the absence or a very low grade of hidden athero in the conduit vessel (PB < 33%) has excellent negative predictive power (100%) in ruling out this phenomenon. All this serves to explain that this phenomenon of slow contrast runoff is due to a baseline vasoconstriction of the microcirculation, possibly related to an endothelium-dependent coronary microvascular dysfunction, expression of the atherosclerosis involvement of the microcirculation.

Coronary flow reserve. We have demonstrated that in SCFph patients, the CFR is not significantly reduced compared to the control group. That is not totally unexpected since the CFR is used to explore the maximum vasodilating capability of the microcirculation using an endothelium-independent mechanism. By means of appropriate stimulation, we can verify the integrity of the microcirculation in terms of gross abnormalities including arteriolar thickening, perivascular accumulations of connective tissue, and capillary microaneurysms, as described in diabetic patients [14]. Even major arteriolar abnormalities have been described in patients with severe hypertrophy and as sequelae of myocardial infarction scarring, with rarefaction of the microvessel network [15]. But in SCFph patients the subtending derangement is the resting increase of the microcirculatory resistance due to an isolated arteriolar endothelium-dependent dysfunction [16] without any more extensive involvement of the interstitium, the arteriolar smooth muscle, etc. However, an isolated endothelium-related arteriolar dysfunction cannot be properly explored by the coronary flow reserve, which acts through an endothelium-independent mechanism. To investigate this, a specific agent that acts on the endothelium is needed such as acetylcholine, or better still, the cold pressure test in order to induce a maximal endothelium-dependent vasodilation. A typical effect of major microvascular endothelium-dependent dysfunction is a vasoconstriction at rest that is spotted in positron emission tomography (PET) studies as an abnormally dishomogeneous perfusion at rest [17]. This kind of abnormality occurs because the constant production of vasodilator (and antithrombotic) factors (in particular, Nitric Oxide [NO]) by the endothelium is impaired, causing this dishomogeneous vasoconstriction of the microcirculation [16,17]. Characteristically, this kind of resting perfusion abnormality disappears during maximally induced vasodilation with an endothelium-independent mechanism, indicating a functional abnormality only of the microvascular endothelium while sparing the smooth muscle arteriolar media layer and the interstitium. In our opinion, this latter situation could apply to the SCFph. According to this interpretation, in patients with the SCFph, the resistance is abnormally high at rest, as described in previous reports. In one invasive study [4], microvascular resistances at rest (mean transit time×distal pressure) were significantly higher in the SCFph group (SCFph: 104 ± 31 versus 53 ± 27, *p* < 0.01). However, patients showed normal responsiveness to vasodilators (bolus injection of papaverine 15 mg); this difference was abolished after the induction of hyperemia (SCFph group: 34 ± 22; control: 22 ± 15, *p* = ns). Hence, the CFR was similar in the SCFph group and control. Further evidence of a dynamically increased coronary microvascular tone has been provided by Mangieri and colleagues [18], who demonstrated angiographic resolution of the SCFph with the arteriolar vasodilator dipyridamole but not with nitrates. 

The same applied in our SCFph patients (Figure 3) that had a normal response to endothelium-independent vasodilators and a similar CFR to that of the control group, proving that in these patients, the microcirculation imbalance is purely based on an endothelial microvascular dysfunction that increases resistance and reduces perfusion only at rest. 

In another study, however, the CFR was lower in the SCFph group [4]. This discrepancy is likely related mainly to the fact that in that study, a single dose of dipyridamole was used. Single dose dipyridamole has a rather weak vasodilating effect, certainly weaker than the vasodilation exerted by adenosine or papaverine used in this and in the previously cited study, respectively, which showed a good CFR in the SCFph group [3]. Accordingly, in a study of validation of CFR with transthoracic-enhanced Doppler [6], to better match the maximal vasodilation of the invasive study using adenosine as vasodilator, we were forced to use not a single but a double dose of dipyridamole (0.54 mg/kg IV over 4 min, followed by 4 min of no dose and then a further 0.28 mg/kg over 2 min).

The potency of the vasodilation could be critical in subjects with a certain degree of microvascular constriction at rest. In fact, even though adenosine is an endothelium-independent vasodilator, it also has a minimal late mechanism (20% of its total effect) that is based on a flow-mediated endothelium-dependent mechanism [19]. The preferential dilation of the downstream small arterioles to adenosine triggers an increase in flow and a decrease in pressure at upstream vessels. The increased flow activates the shear-sensitive mechanism of the upstream large arterioles and further enhances the flow. This hemodynamic interaction contributes up to approximately 20% of the adenosine-induced flow increase and reduces the adenosine-induced pressure drop. Therefore, a weak vasodilator of the small arterioles (like dipyridamole), with a possibly even weaker effect due to the presence of baseline arteriolar constriction, also reduces the added secondary flow-mediated effect, significantly reducing the total vasodilation, the hyperemic flow, and the reserve. The lack of this upstream large arteriolar vasodilation through the shear stress and thus with an endothelium-dependent mechanism could also explain the trend of lower hyperemic velocity in the SCFph than in the control. However, overall, adenosine maximal effect is modestly affected by this purely endothelium-dependent mechanism. 

Atherosclerosis of the microcirculation. We found that in the SCFph patients, the abnormal resting vasoconstrictive tone, previously invasively documented by Fineschi [3], is associated with hidden epicardial athero, and that the absence of hidden athero rules out the SCFph (Figure 4). In short, the driver of this resting microcirculatory dysfunction appears to be related to the penetration of atherosclerosis into the microcirculation. 

Since we have documented that the SCFph does not occur in the absence of hidden atherosclosis, we believe that a slow runoff at rest in the absence of other causes of augmented microvascular resistance, such as embolization in the microcirculation, is due to atherosclerotic etiologic factors acting on the microcirculation.

Coronary atherosclerosis, in fact, impairs not only endothelium-dependent epicardial arterial dilation but also impairs arteriolar vasodilation and flow increment after the infusion of acetylcholine or other vasodilators, or during the cold pressure test. In the arteriolar compartment, the effect of atherogenic factors [20] such as oxidized cholesterols (in particular, low-density lipoproteins [LDL] and microparticles), elevated homocysteine, metabolic syndrome and diabetes, pesticides, heavy metals, etc., to name just a few, does not create atherogenic plaques but exerts a damaging effect, mainly on endothelial cells [20]. Microscopic evaluation shows vacuoles in the endothelial cells of the microcirculation [21]. However, what is more important is that the constant production of vasodilating substances such as NO becomes impaired [22]. This microvascular endothelial damage has been reproduced by a high cholesterol diet alone in cholesterol-fed rabbits [23]. The final effect is that arteriolar vasoconstriction prevails [16]. Our data in this respect confirm the few data reported in the literature [24,25]; in these two studies, the strong association between epicardial hidden atherosclerosis and the SCFph was confirmed, although they lacked a control group. In addition, they found a hyperemic pressure gradient in coronaries by fractional flow reserve (FFR) but CFR was not measured, and it was speculated that the atherosclerotic burden could have played a role in creating the SCFph. In our experience, the FFR gradient is only an expression of diffuse atherosclerosis, which exerts its effect only during hyperemia by creating a longitudinal base to apex perfusion gradient visible with PET [26], reducing the hyperemic flow due to the diffuse epicardial athero [27] and resistance, consequently impairing the CFR [28]. The SCFph, rather, is a resting phenomenon modulated by the microcirculation. However, the two conditions may coexist.

Previous studies. Previous studies that have attempted to study the SCFph, especially through the evaluation of CFR, are limited mainly because of the limited number of enrolled patients since these studies were mainly invasive, thus clearly restricting recruitment [3,4,18,29,30,31,32,33,34]. Moreover, the CFR procedures were old and consequently limited [32,35]. In this study, thanks to the high feasibility [8], reliability [7,36,37], and total non-invasiveness of the E-Doppler TTE in blood flow Doppler recording in coronaries, CFR was attained in a conspicuous number of patients in order to draw more meaningful conclusions regarding the role of CFR in this slow runoff phenomenon. In addition, and even more importantly, a certain light has been shed on the etiology of this phenomenon, thanks to the IVUS study, albeit only in a subgroup of patients. 

Clinical Implications. The SCFph is not without clinical consequences, as reported [38]. Angina is the major clinical complaint, confirmed in our SCFph series. However, the coronary endothelium dysfunction that affected the SCFph angiographic finding is a major clinical problem, since it is a condition marking the progression and instability of the coronary artery disease [17]. The majority of patients with angina, in the absence of obstructive coronary artery disease or the SCFph, have occult coronary abnormalities. A comprehensive invasive assessment of these patients at the time of coronary angiography (IVUS, CFR, and acetylcholine test for endothelium-dependent function) provides important diagnostic information that may affect treatment and outcomes [39]. In particular, based on our data, IVUS should become routine in subjects with no angiographic stenosis, angina, and the slow coronary flow phenomenon. Alternatively, a functional evaluation of endothelium-dependent arteriolar function can be obtained after catheterization by executing a cold pressure test during flow velocity monitoring in the distal LAD by E-Doppler TTE; in this way, x-ray exposure and contrast risk can be reduced. The cold pressure test executed with echocardiography was first proposed with a transesophageal Doppler [40], but can now be performed with an E-Doppler TTE. It is extremely effective with a feasibility of 100% and has extremely high reproducibility, as recently demonstrated in our lab (unpublished data). A surrogate approach for coronary endothelial function is flow-mediated dilatation in the brachial artery. However, the brachial artery response is not a good predictor of coronary endothelial function in individual patients [41], as recently reported. The endothelial function in fact can be modeled by hemodynamic forces and shear stress, which are profoundly different in the brachial artery and coronary territory [20]. Because such forces can influence endothelial cells’ gene expression, even the ensuing endothelial function is different; therefore, cells may react differently to endothelial offenders such as oxidized LDL. 

Limitations. Some limitations of this study must be mentioned.

Only a limited number of patients underwent IVUS, so our data on the association of hidden athero with SCFph must be considered only preliminary. 

The injection was given manually; a different strength in pushing the contrast into the coronaries could affect, albeit minimally, the velocity of contrast progression in the coronaries according to the amount of intraarterial pressure increase distal to the catheter tip caused by the injection [31]. 

A challenge test (cold pressure, acetylcholine etc.) to explore the arteriolar endothelium-dependent function was not performed. In the future, this test, especially if non-invasive (E-Doppler TTE), may become the cornerstone for the evaluation of coronary artery disease, since in cases of scarce response, it indicates instability and a tendency toward the progression of coronary artery disease.

## 5. Conclusions

The slow coronary flow phenomenon in patients with angina and no angiographic stenosis is not associated to an impaired CFR. The SCFph seems to be determined by a coronary endothelium-dependent arteriolar dysfunction with microvascular constriction at rest caused by the atherosclerotic involvement of the coronary microcirculation.

## Figures and Tables

**Table 1:** Hemodynamics and flow velocity parameters in the distal LAD by E-Doppler TTE during CFR assessment in patients with normal and slow runoff.

	Runoff Characteristics		*p* Value
	normal	SCFph	
**Hemodynamics**			
**Rest SBP (mmHg)**	127 ± 16	118 ± 13	*<0.05*
**Rest DBP (mmHg)**	70 ± 9	67 ± 8	*ns*
**rest HR (b/m)**	64 ± 64	64 ± 8	*ns*
**Adenosine SBP (mmHg)**	128 ± 18	123 ± 17	*ns*
**Adenosine DBP (mmHg)**	70 ± 10	67 ± 10	*ns*
**Adenosine HR (b/m)**	75 ± 17	75 ± 13	*ns*
***Control velocity (cm/s)***			
**MSV**	16 ± 4	15 ± 4	*ns*
**PSV**	20 ± 5	18 ± 5	*ns*
**MDV**	25 ± 7	23 ± 7	*ns*
**PDV**	33 ± 9	32 ± 13	*ns*
***Hyperemic velocity (cm/s)***			
**MSV**	46 ± 23	36 ± 9	*ns*
**PSV**	56 ± 21	46 ± 13	*ns*
**MDV**	71 ± 23	62 ± 12	*ns*
**PDV**	93 ± 27	82 ± 17	*=0.07*
**LAD CFR for PDV**	2.90 ± 0.8	2.79 ± 0.8	*ns*
**LAD CFR for MDV**	2.22 ± 0.6	2.12 ± 0.6	*ns*

SBP indicates systolic blood pressure; DBP, diastolic blood pressure; HR, heart rate; PDV, peak diastolic velocity; MDV, mean diastolic velocity; PSV, peak systolic velocity; MSV, mean systolic velocity; and SCFph = slow coronary runoff.

**Table 2:** Baseline characteristics of the study population according to runoff characteristics.

Variable	Runoff Characteristics		*p* Value
	Normal (*n* = 100)	Slow (*n* = 24)	
Age (years)	50.53 ± 12.35	47.70 ± 13.28	*=0.3*
Male-# pts (%)	67 (67%)	15 (62.5%)	*=0.7*
BMI	27.70 ± 5.20	27.79 ± 6.87	*=0.7*
Hypertension-# pts (%)	71 (71%)	17 (70.8%)	*=1.0*
Diabetes mellitus-# pts (%)	15 (15%)	2 (8.3%)	*=0.4*
Glycemia (mg/dL)	104.89 ± 27.33	110.48 ± 48.55	*=0.4*
Total cholesterol (mg/dL)	167.26 ± 43.49	182.96 ± 40.27	*=0.1*
HDL (mg/dL)	50.24 ± 16.86	46.65 ± 9.31	*=0.3*
LDL (mg/dL)	94.56 ± 35.35	113.28 ± 43.24	***=0.03***
Triglycerides (mg/dL)	115.53 ± 62.81	133.57 ± 86.55	*=0.2*
Smoking-# pts (%)	15 (15%)	6 (25%)	*=0.2*
Angina-# pts (%)	32 (32%)	12 (50%)	*=0.4*
NYHA II-# pts (%)	21 (65.6%)	6 (85.7%)	*-*
NYHA III-# pts (%)	10 (31.3%)	1 (14.3%)	*-*
NYHA IV-# pts (%)	1 (3.1%)	0 (0%)	*=0.1*
DCM-# pts (%)	24 (24%)	4 (16.7%)	*=0.4*
ASCVD risk score (%)	12.16 ± 9.52	10.28 ± 11.98	*=0.6*
LVEF (%)	55 ± 13	59 ± 13	*=0.2*
Statins-# pts (%)	70 (70%)	14 (58%)	*=0.3*
Betablockers-# pts (%)	21(30%)	4 (23%)	*=0.6*
Antithrombotics-# pts (%)	51 (52%)	11 (45%)	*=0.5*
ACEi-# pts (%)	75 (75%)	17 (71%)	*=0.7*
Diuretics-# pts (%)	14 (14%)	1 (4%)	*=0.2*

HDL = high-density lipoprotein; LDL = low-density lipoprotein; BMI= Body Mass Index; NYHA = New York Heart Association; DCM = dilated cardiomyopathy; ASCVD = atherosclerotic cardiovascular disease; LVEF = left ventricular ejection fraction; ACEi = Angiotensin converting enzyme inhibitor; pts= patients; in bold the *p* value that was significant.

## Data Availability

Not applicable.

## References

[1] Tambe A.A., Demany M.A., Zimmerman H.A., Mascarenhas E. (1972). Angina pectoris and slow flow velocity of dye in coronary arteries–A new angiographic finding. Am. Heart J..

[2] Wang X., Nie S.P. (2011). The coronary slow flow phenomenon: Characteristics, mechanisms and implications. Cardiovasc. Diagn. Ther..

[3] Fineschi M., Bravi A., Gori T. (2008). The “slow coronary flow” phenomenon: Evidence of preserved coronary flow reserve despite increased resting microvascular resistances. Int. J. Cardiol..

[4] Erdogan D., Caliskan M., Gullu H., Sezgin A.T., Yildirir A., Muderrisoglu H. (2007). Coronary flow reserve is impaired in patients with slow coronary flow. Atherosclerosis.

[5] Rossen J.D., Quillen J.E., Lopez A.G., Stenberg R.G., Talman C.L., Winniford M.D. (1991). Comparison of coronary vasodilation with intravenous dipyridamole and adenosine. J. Am. Coll. Cardiol..

[6] Caiati C., Montaldo C., Zedda N., Bina A., Iliceto S. (1999). New noninvasive method for coronary flow reserve assessment-Contrast-enhanced transthoracic second harmonic echo Doppler. Circulation.

[7] Caiati C., Montaldo C., Zedda N., Montisci R., Ruscazio V., Lai G., Cadeddu J., Meloni L., Iliceto S. (1999). Validation of a new noninvasive, method (contrast-enhanced transthoracic second harmonic echo Doppler) for the evaluation of coronary flow reserve-Comparison with intracoronary Doppler flow wire. J. Am. Coll. Cardiol..

[8] Caiati C., Zedda N., Montaldo C., Montisci R., Iliceto S. (1999). Contrast-enhanced transthoracic second harmonic echo Doppler with adenosine: A noninvasive, rapid and effective method for coronary flow reserve assessment. J. Am. Coll. Cardiol..

[9] Caiati C., Lepera M.E., Pollice P., Iacovelli F., Favale S. (2020). A new noninvasive method for assessing mild coronary atherosclerosis: Transthoracic convergent color Doppler after heart rate reduction. Validation vs. intracoronary ultrasound. Coron. Artery Dis..

[10] Gibson C.M., Cannon C.P., Daley W.L., Dodge J.T., Alexander B., Marble S.J., McCabe C.H., Raymond L., Fortin T., Poole W.K. (1996). TIMI frame count: A quantitative method of assessing coronary artery flow. Circulation.

[11] MacAlpin R.N., Abbasi A.S., Grollman J.H., Eber L. (1973). Human coronary artery size during life. A cinearteriographic study. Radiology.

[12] Mintz G.S., Nissen S.E., Anderson W.D., Bailey S.R., Erbel R., Fitzgerald P.J., Pinto F.J., Rosenfield K., Siegel R.J., Tuzcu E.M. (2001). American College of Cardiology Clinical Expert Consensus Document on Standards for Acquisition, Measurement and Reporting of Intravascular Ultrasound Studies (IVUS). A report of the American College of Cardiology Task Force on Clinical Expert Consensus Documents. J. Am. Coll. Cardiol..

[13] Reis S.E., Holubkov R., Lee J.S., Sharaf B., Reichek N., Rogers W.J., Walsh E.G., Fuisz A.R., Kerensky R., Detre K.M. (1999). Coronary flow velocity response to adenosine characterizes coronary microvascular function in women with chest pain and no obstructive coronary disease. Results from the pilot phase of the Women’s Ischemia Syndrome Evaluation (WISE) study. J. Am. Coll. Cardiol..

[14] Munzel T., Daiber A., Ullrich V., Mulsch A. (2005). Vascular consequences of endothelial nitric oxide synthase uncoupling for the activity and expression of the soluble guanylyl cyclase and the cGMP-dependent protein kinase. Arter. Thromb. Vasc. Biol..

[15] Mohammed S.F., Hussain S., Mirzoyev S.A., Edwards W.D., Maleszewski J.J., Redfield M.M. (2015). Coronary microvascular rarefaction and myocardial fibrosis in heart failure with preserved ejection fraction. Circulation.

[16] Schindler T.H., Schelbert H.R., Quercioli A., Dilsizian V. (2010). Cardiac PET imaging for the detection and monitoring of coronary artery disease and microvascular health. JACC Cardiovasc. Imaging.

[17] Gould K.L., Ornish D., Scherwitz L., Brown S., Edens R.P., Hess M.J., Mullani N., Bolomey L., Dobbs F., Armstrong W.T. (1995). Changes in myocardial perfusion abnormalities by positron emission tomography after long-term, intense risk factor modification. JAMA.

[18] Mangieri E., Macchiarelli G., Ciavolella M., Barilla F., Avella A., Martinotti A., Dell’Italia L.J., Scibilia G., Motta P., Campa P.P. (1996). Slow coronary flow: Clinical and histopathological features in patients with otherwise normal epicardial coronary arteries. Cathet. Cardiovasc. Diagn..

[19] Liao J.C., Kuo L. (1997). Interaction between adenosine and flow-induced dilation in coronary microvascular network. Am. J. Physiol..

[20] Caiati C. (2019). Contrast-Enhanced Ultrasound Reveals That Lipoprotein Apheresis Improves Myocardial But Not Skeletal Muscle Perfusion. JACC Cardiovasc. Imaging.

[21] Sellke F.W., Armstrong M.L., Harrison D.G. (1990). Endothelium-dependent vascular relaxation is abnormal in the coronary microcirculation of atherosclerotic primates. Circulation.

[22] Tousoulis D., Kampoli A.M., Tentolouris C., Papageorgiou N., Stefanadis C. (2012). The role of nitric oxide on endothelial function. Curr. Vasc. Pharmacol..

[23] Osborne J.A., Siegman M.J., Sedar A.W., Mooers S.U., Lefer A.M. (1989). Lack of endothelium-dependent relaxation in coronary resistance arteries of cholesterol-fed rabbits. Am. J. Physiol..

[24] Cin V.G., Pekdemir H., Camsar A., Ciçek D., Akkus M.N., Parmaksýz T., Katýrcýbaý T., Döven O. (2003). Diffuse intimal thickening of coronary arteries in slow coronary flow. Jpn. Heart J..

[25] Pekdemir H., Cin V.G., Ciçek D., Camsari A., Akkus N., Döven O., Parmaksiz H.T. (2004). Slow coronary flow may be a sign of diffuse atherosclerosis. Contribution of FFR and IVUS. Acta Cardiol..

[26] Gould K.L., Nguyen T., Johnson N.P. (2019). Integrating Coronary Physiology, Longitudinal Pressure, and Perfusion Gradients in CAD: Measurements, Meaning, and Mortality. J. Am. Coll. Cardiol..

[27] Gould K.L., Johnson N.P. (2018). Coronary Physiology Beyond Coronary Flow Reserve in Microvascular Angina: JACC State-of-the-Art Review. J. Am. Coll. Cardiol..

[28] Caiati C., Siena P., Iacovelli F., Piscitelli L., Pollice P., Favale S., Lepera Mario E. (2021). Assessing Diffuse Coronary Atherosclerosis in Subjects with Impaired Coronary Flow Reserve but no Angiographic Critical Stenosis. A Transthoracic Enhanced Color Doppler Echocardiographyc Study. J. Am. Coll. Cardiol. Found..

[29] Alvarez C., Siu H. (2018). Coronary Slow-Flow Phenomenon as an Underrecognized and Treatable Source of Chest Pain: Case Series and Literature Review. J. Investig. Med. High Impact Case Rep..

[30] Azzarelli S., Grasso C., Galassi A.R., Tamburino C. (2005). Coronary slow flow phenomenon: Description of three cases evaluated with myocardial perfusion scintigraphy. Ital. Heart J. Off. J. Ital. Fed. Cardiol..

[31] Levin D.C., Phillips D.A., Lee-Son S., Maroko P.R. (1977). Hemodynamic changes distal to selective arterial injections. Investig. Radiol..

[32] Van Lierde J., Vrolix M., Sionis D., De Geest H., Piessens J. (1991). Lack of evidence for small vessel disease in a patient with “slow dye progression” in the coronary arteries. Cathet. Cardiovasc. Diagn.

[33] Chaudhry M.A., Smith M., Hanna E.B., Lazzara R. (2012). Diverse spectrum of presentation of coronary slow flow phenomenon: A concise review of the literature. Cardiol. Res. Pract..

[34] Beltrame J.F., Limaye S.B., Horowitz J.D. (2002). The coronary slow flow phenomenon–A new coronary microvascular disorder. Cardiology.

[35] Beltrame J.F., Limaye S.B., Wuttke R.D., Horowitz J.D. (2003). Coronary hemodynamic and metabolic studies of the coronary slow flow phenomenon. Am. Heart J..

[36] Knuuti J., Wijns W., Saraste A., Capodanno D., Barbato E., Funck-Brentano C., Prescott E., Storey R.F., Deaton C., Cuisset T. (2019). 2019 ESC Guidelines for the diagnosis and management of chronic coronary syndromes. Eur. Heart J..

[37] Crea F., Montone R.A., Rinaldi R. (2021). Pathophysiology of Coronary Microvascular Dysfunction. Circ. J..

[38] Zhu X., Shen H., Gao F., Wu S., Ma Q., Jia S., Zhao Z., Tong S., Zhang Z., Zhou Y. (2019). Clinical Profile and Outcome in Patients with Coronary Slow Flow Phenomenon. Cardiol. Res. Pract..

[39] Lee B.K., Lim H.S., Fearon W.F., Yong A.S., Yamada R., Tanaka S., Lee D.P., Yeung A.C., Tremmel J.A. (2015). Invasive evaluation of patients with angina in the absence of obstructive coronary artery disease. Circulation.

[40] Chandraratna P.A., Nimalasuriya A.R., Vlachonassios K.D., Mathews S.J., Kedes W., Marwah O.S., Saad M. (1999). Usefulness of the response of flow velocity in the left anterior descending coronary artery to the cold pressor test for evaluating endothelium-dependent vascular relaxation in the coronary microvasculature by transesophageal echocardiography in subjects with angiographically normal coronary arteries. Am. J. Cardiol..

[41] Ganz P., Vita J.A. (2003). Testing endothelial vasomotor function: Nitric oxide, a multipotent molecule. Circulation.

